# MicroRNAs in Cancer: A Historical Perspective on the Path from Discovery to Therapy

**DOI:** 10.3390/cancers7030842

**Published:** 2015-07-27

**Authors:** Esteban A. Orellana, Andrea L. Kasinski

**Affiliations:** 1 Department of Biological Sciences, Bindley Bioscience Center, Purdue University, 1203 West State Street, West Lafayette, IN 47907, USA; 2 Purdue University Interdisciplinary Life Science Program (PULSe), West Lafayette, IN 47907, USA

**Keywords:** microRNA, history, cancer, therapeutics

## Abstract

Recent progress in microRNA (miRNA) therapeutics has been strongly dependent on multiple seminal discoveries in the area of miRNA biology during the past two decades. In this review, we focus on the historical discoveries that collectively led to transitioning miRNAs into the clinic. We highlight the pivotal studies that identified the first miRNAs in *Caenorhabditis elegans* to the more recent reports that have fueled the quest to understand the use of miRNAs as markers for cancer diagnosis and prognosis. In addition, we provide insights as to how unraveling basic miRNA biology has provided a solid foundation for advancing miRNAs, such as miR-34a, therapeutically. We conclude with a brief examination of the current challenges that still need to be addressed to accelerate the path of miRNAs to the clinic: including delivery vehicles, miRNA- and delivery-associated toxicity, dosage, and off target effects.

## 1. Introduction

The functional importance of microRNAs (miRNAs) has been extensively reported not only in normal biological processes but also in human diseases such as cancer. Since their discovery, twenty-two years ago, the knowledge on miRNAs and cancer has been increasing exponentially (>18,500 hits in PubMed accessed in June 2015).

In this review, we highlight historical discoveries that collectively have contributed to the emerging field of miRNA therapeutics. Herein, we have divided this review into four main stages: early history, miRNA basic biology, miRNA misregulation in cancer, and miRNA transition to therapy.

## 2. Early History: MiRNA Discovery

The history of microRNAs started with basic research in microscopic worms. In the mid 1970s, Sydney Brenner was studying neural development in *Caenorhabditis elegans* and during his screening of mutant worms isolated the first nematode that had a developmental timing defect (heterochronic mutant) named *lin-4* (cell lineage abnormal) [1,2]. This particular discovery has led to insights of substantial importance to human biology and disease. In the early 1980s, Robert Horvitz who worked as a postdoctoral research fellow in the Brenner lab along with John Sulston, a staff scientist also in the same laboratory, characterized *lin-4* during the inspiring quest to understand how the temporal development pattern in animals is controlled [3]*.* It was determined that the abnormal lineage was a result of a null mutation (*e912*) in the heterochronic gene *lin-4* that caused abnormal temporal development [3,4]. Individual worms bearing the *lin-4(e912)* mutation ultimately develop an abnormal adult phenotype; the worms reiterate late larval stage cell fates and thus are incapable of laying eggs [3,4].

In 1984, Victor Ambros, a postdoctoral fellow in the Horvitz lab, worked to characterize heterochronic mutants of *C. elegans,* and found another heterochronic mutant lineage in null *lin-14* mutants, that presented an opposing phenotype to *lin-4* (e912) [5], animals skipped early fates and instead produced later fates precociously. Later, in 1987, Horvitz and colleagues, Edwin Ferguson and Paul Sternberg, reported that null mutations in the *lin-14* gene reversed the phenotype seen in *lin-4* loss of function [6]. These intriguing observations suggested an epistatic interaction in which *lin-4* negatively regulates *lin-14*. The initial assumption was that the gene product of *lin-4* was most likely a protein acting as a negative regulator of *lin-14*; however, this would be proven incorrect during the following years. Victor Ambros and Gary Ruvkun, both trained in the Horvitz lab, now as principal investigators in their own laboratories continued their research characterizing the *lin-4*–*lin-14* interaction.

**Figure 1:**
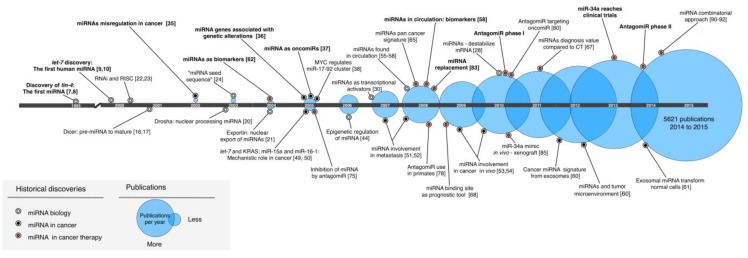
Selected historical discoveries that collectively led to transitioning miRNAs into the clinic. The selected hallmarks are divided into miRNA biology, involvement in cancer, and advances in miRNA-based cancer therapeutics. The circles represent the number of publications per single year (PubMed query: miRNA AND cancer; accessed: June 2015). CT: computed tomography.

In 1993, two independent and mutually reinforcing studies were published in the same issue of *Cell* as a result of sharing ideas and unpublished results. First, Rosalind Lee and Rhonda Feinbaum working at Ambros’ lab demonstrated that the genomic locus that contained *lin-4* did not encode a protein [7]. Instead, Ambros and colleagues, identified two small non-coding *lin-4* transcripts of 22 and 61 nt respectively [7]. In the other publication, Ruvkun along with Bruce Wightman and Ilho Ha identified seven elements in the 3′ untranslated region (UTR) of *lin-14* that had sequence complementarity to the *lin-4* small RNAs [8]. These two independent studies had discovered a novel mechanism in which *lin-4* mediated its effects on *lin-14* through a posttranscriptional mechanism via an antisense RNA duplex interaction [7,8]. The work that initiated with the identification of heterochronic *C. elegans* mutants led to the unexpected discovery of an entirely new type of regulatory mechanism mediated by a non-coding RNA and established a new paradigm that challenged the central dogma in biology (see Figure 1 for an extensive view of the progression of the miRNA field beginning with the work of Ambros and Ruvkun).

For seven years there were no signs that similar non-coding RNA regulatory mechanisms existed in *C. elegans* or any other metazoan. That changed in 2000, when the Ruvkun laboratory reported they had identified a second heterochronic gene in nematodes, *lethal-7* (*let-7*), that did not encode a protein product but rather produced a 22 nt RNA [9,10]. Similarly to *lin-4,* the *let-7* regulatory RNA was essential for cell fate transitions from the larval to adult stages. In February of 2000, Brenda Reinhart and Frank Slack in Ruvkun’s lab demonstrated that loss of *let-7* causes transformations in which larval cellular fates are reiterated, while increased levels of *let-7* leads to omission of larval-specific events [9]. In April of the same year, Frank Slack and colleagues discovered that *let-7* activation during late larval stages regulates the nematode larval-adult transition by downregulating its target LIN-41*,* which in turn negatively regulates LIN-29, a transcription factor that controls of adult specification [10]. Only a few short months later, Amy Pasquinelli and her colleagues in the Ruvkun laboratory reported that they had identified *let-7* RNA homologues in multiple animal species including humans and other model organisms [11]. The developmental regulation mediated by *let-7* was shown to be conserved among other species including *Drosophila* and zebrafish, suggesting that the mechanism mediated by the small non-coding RNA *let-7* was conserved through evolution [11]. 

By the year 2000, the Ambros and Ruvkun laboratories had discovered the two founding members of a family of small non-coding RNAs, now called microRNAs (miRNAs), a rather large family with members not only in nematodes but also widespread in insects, plants and mammals [11,12,13,14,15]. These seminal discoveries were followed in 2001 by three independent studies that provided further support that miRNAs were indeed conserved through evolution, and that other miRNAs were present in invertebrates and mammals implying that miRNA functions could be a general gene regulatory mechanism in eukaryotes [12,13,14].

## 3. MiRNA Basic Biology

### 3.1. MiRNA Biogenesis

In early 2000s, these newly discovered small RNAs started to be recognized as important members of the gene regulatory arsenal within a cell. However, their biogenesis was still obscure. The detection of miRNA transcripts of different sizes (22 and 61 nt) suggested that the 22 nt transcripts originated from larger precursor transcript [7,9,11,16]. In 2001, a mechanism for miRNA processing was documented for the first time by Mello, Ruvkun and Zamore. Mechanistically, the immature form (pre-miRNA, 60 to 110 nt) was processed by the cytoplasmic enzyme Dicer into a short miRNA duplex (~20 nt, dsRNA) [16,17]. Very shortly thereafter, it was demonstrated that some miRNAs are transcribed as a longer primary transcripts (pri-miRNAs), which are typically capped and polyadenylated [18]. These pri-miRNAs are processed into a mature form through a controlled and sequential process [19]. In 2003, Lee and colleagues identified a human nuclear RNase III termed Drosha that was responsible for initiation of the miRNA maturation process [20]. The authors showed that Drosha mediated nuclear processing of pri-miRNA transcripts into 70 nt pre-miRNAs [20]. Altogether these studies demonstrated that miRNA maturation was a sequential and compartmentalized process: (i) generation of pre-miRNAs from pri-miRNAs in the nucleus by Drosha and (ii) processing of pre-miRNAs into mature miRNAs in the cytoplasm by Dicer. The fact that the two sequential steps occurred in two subcellular localizations suggested that pre-miRNAs had to undergo nuclear export, but the underlying mechanism was still unknown. That changed in 2004, when Elsebet Lund and colleagues reported that the nuclear karyopherin exportin-5 mediates efficient export of pre-miRNAs from the nucleus into the cytoplasm [21].

### 3.2. MiRNA Biological Function

The knowledge necessary to understand how these miRNAs work was acquired from the study of the RNA interference (RNAi) phenomenon initially identified in plants. RNA silencing in plants was determined to be mediated by dsRNAs of ~21–23 nt (small interfering RNAs, siRNAs) with sequence complementarity to the target mRNA which triggers mRNA cleavage [22]. This phenomenon, reported in 2000, was followed in the same year with the discovery of the mechanism leading to mRNA degradation. Scott Hammond and colleagues demonstrated that RNAi targeted mRNA degradation through the formation of a sequence-specific nuclease that they termed RISC (RNA-induced silencing complex). Single strand RNA species derived from both miRNAs and siRNAs (dsRNAs) were determined to be the signature component of RISC [23].

In regards to miRNA target specificity, in 2003 an approach was described to predict mammalian miRNA targets based on sequence pairing to the 5′ end of the miRNA, particularly nucleotides 2–8, which was referred as the “miRNA seed sequence” [24]. This came from the elucidation that miRNA-mRNA binding identified in this study was based on perfect complementarity only within the seed region; the remaining sequence of the miRNA paired imperfectly with the target. From a historical perspective, the study and identification of miRNA gene targets was limited to the identification of the complementary sequence of the miRNA seed in the 3′ UTR of mRNAs. That changed in 2007 when Robin Lytle and colleagues published the first report suggesting that miRNAs could also bind to the 5′ UTR and exert gene repression [25]. The following findings showed that miRNAs could also bind to the coding sequence (CDS) of their target genes [26]. While the “seed-binding” hypothesis is incorporated into many miRNA-mRNA binding algorithms and is further used to classify miRNAs into families, recent studies suggest seed-independent binding. In 2013 Aleksandra Helwak and colleagues developed an elegant experimental approach to identify targets of miRNAs *in vivo* and reported that indeed most miRNA-mRNA interactions include the seed region; however, more that 60% of seed interactions contain mismatched nucleotides and bulges, suggesting seed-independent interactions [27]. Even more interesting they describe a data set of more than 18,000 miRNA-mRNA interactions in which ~42% of interactions occur in the CDS, ~23% in the 3′ UTR, and ~4% in the 5′ UTR, further supporting the hypothesis that miRNAs could control protein production by binding targets beyond the 3′ UTR [27].

MiRNAs were primarily associated with negative posttranscriptional regulation of gene expression, which could be achieved by blocking translation or causing mRNA degradation, but the contributions of each mechanism remained obscure. In 2010, Huili Guo and colleagues found that destabilization of mRNA account for more than 84% of the decreased protein output [28]. Far less understood functions of miRNAs have also been reported. For instance, miRNAs have been shown to activate protein translation in certain cellular contexts [29,30,31], could act as a decoy for regulatory proteins [32] and could play a role in RNA crosstalk [33,34].

## 4. MiRNA Misregulation in Cancer

From all the insights gained from the study of miRNAs in *C. elegans* development it was evident that tight control of miRNA expression and processing was crucial to sustain normal cellular functions and homeostasis. Indeed, in 2002, only two years after the discovery of the first human miRNA (*let-7*), the first report suggesting the involvement of miRNAs in cancer was published. George Calin and colleagues, working in Carlo Croce’s laboratory attempted to find tumor suppressor genes within a chromosomal region (13q14) that is often deleted in patients with B cell chronic lymphocytic leukemia (B-CLL) [35]. Instead of finding a canonical tumor suppressor gene that encodes a protein, they found that this region harbored two miRNA genes, *mir-15a* and *mir-16-1* [35]. Indeed, for the first time in the brief history of miRNAs researchers had demonstrated the consistent involvement of two miRNAs in B-CLL, where both miRNAs are either deleted or downregulated, suggesting their potential role as tumor suppressors [35]. Later, in a follow-up study, the same research group mapped 98 miRNAs to locations previously reported to be associated with genetic alterations either involved in cancer or contained in fragile sites of the genome [36]. Similarly, the first indication that miRNAs may also function as oncogenes was reported by He, Thomson and colleagues in 2005. In the first of its kind experiment, overexpression of the polycistronic transcript encoding the miR-17~92 cluster *in vivo*, which is overexpressed in patients with B-CLL, demonstrated that these miRNAs could enhance lymphoma in a mouse model induced by the *c-Myc* oncogene [37]. These oncogenic miRNAs have since been termed oncomiRs. These seminal studies provided several indications of the involvement of miRNAs in human cancers and paved the path to understand the importance of miRNAs in tumorigenesis and other diseases.

Although, the biological importance of miRNAs started to become apparent by mid 2000s, still very little was known about the regulatory mechanisms that control miRNA expression. In 2005, Kathryn O'Donnell and colleagues reported that the transcription factor c-MYC, one of the most common oncogenes in human neoplasms, was able to regulate the expression of the miR-17~92 cluster [38]. Similarly, p53, the most commonly mutated tumor suppressor gene in human cancers, was identified as a transcriptional regulator of the miR-34 family of miRNAs. Five independent groups identified that the three miR-34 genes, *mir-34a*, *mir-34b*, and *mir-34c*, the later two expressed from one transcript, were transcriptionally regulated by p53 and that these small RNAs could compensate for some of the tumor suppressive function of p53. Collectively, these studies, revealed for the first time in the vast history of p53 biology, that miRNAs were a core component of the tumor suppressive role of p53 [39,40,41,42,43].

In addition to regulation of miRNA genes by transcription factors, miRNAs were also found to be modulated by epigenetic mechanisms. In 2006, Yoshimasa Saito and colleagues demonstrated that both DNA methylation and histone modification play a role in regulating expression of miRNAs, particularly miR-127. MiR-127 is often silenced in cancer cells; however in the presence of chromatin-modifying drugs (*i.e*., DNA-demethylating agents and histone deacetylase inhibitors) miR-127 is highly induced from its own promoter [44]. A similar situation was observed for miR-34a. While, loss of p53 can lead to reduced expression of miR-34a, *mir-34a* is also regulated by promoter methylation. All tumor types analyzed in the study had appreciable levels of methylation at the *mir-34a* promoter that resulted in diminished miR-34a expression [45]. From 2002 to the present, a myriad of studies have confirmed that miRNA deregulation is a hallmark of cancers as a result of mutations, deletions, amplifications, and dysregulation of transcriptional regulatory cascades and processing machinery (For a detailed review see [46,47]).

In the early 2000s, apart from few exceptions, the roles and functions of most miRNAs were still unknown. In 2003, the first tangible evidence addressing the involvement of a miRNA in controlling cell cycle progression was published. Julius Brennecke and colleagues reported that the *bantam* gene in *Drosophila* encoded a miRNA capable of promoting tissue growth by targeting the pro-apoptotic gene *hid* thus playing a role in cell progression and apoptosis [48]. It was not until 2005 that the first reports of miRNAs’ mechanistic role in cancer came to light. Steven Johnson and colleagues, working in Frank Slack’s laboratory provided evidence that *let-7* targeted the 3′ UTR of RAS, an important oncogene driving multiple cancers [49]. Indeed, the authors demonstrated that the three human *RAS* genes had complementary binding sites for *let-7*, showed that *let-7* could mediate RAS inhibition *in vitro*, and showed the anti-correlation of lower expression of *let-7* with over expression of RAS in lung cancer [49]. In the same year, in another scientific communication from the Croce lab, leading author Amelia Cimmino reported that miR-15a and miR-16-1 negatively regulate the antiapoptotic B cell lymphoma 2 gene (*Bcl2*) causing CLL cells to undergo apoptosis [50]; therefore, contributing the missing functional piece of evidence related to the initial identification of *mir-15a*~*mir-16-1* genomic loss made by the same laboratory in previous years [35].

In 2007–2008, miRNAs were shown to play a dual role in tumor invasion and metastasis. On one hand, miRNAs could promote tumor metastasis, specifically miR-10b, as demonstrated by Li Ma working in Robert Weinberg laboratory [51]. This research group unraveled a mechanism in which miR-10b positively regulates cell migration and invasion in a non-metastatic breast cancer cell line in a multi-step process that ultimately leads to activation of RHOC, a pro-metastatic gene [51]. On the other hand, in 2008 a set of miRNAs capable of suppressing metastasis *in vivo* via ectopic restoration were identified. In brief, Sohail Tavazoie and colleagues described how miR-126 inhibits metastasis by suppressing tumor growth and proliferation whereas miR-335 and miR-206 achieve the same effect by regulating migration and changing morphology [52].

In 2009, the first *in vivo* evidence implicating the overexpression of a miRNA in cancer was communicated. Costinean, Sandhu and other colleagues from the Croce lab reported that miR-155 directly caused a leukemic phenotype in a mouse model overexpressing a miR-155 transgene [53]. The following year the Slack group reported an astonishing finding that a single oncogenic miRNA, miR-21, was enough to cause neoplastic development in a mouse model with no predisposing mutations [54]. Moreover, the authors showed that the tumors decreased in size and survival was restored when miR-21 overexpression was shut down [54]. This evidence suggested that tumors could become addicted to miR-21 in a similar phenomenon to oncogene addiction (oncomiR addiction) [54].

While the elucidation of miRNA involvement in cancer was increasing, early reports in 2008 showed that miRNAs could also be found in circulation [55,56,57,58], but the specific mechanisms conferring their stability or their functional roles were still unknown. The most widely accepted model in the literature was that miRNAs in circulation were protected within nanovesicles (known as exosomes). Later studies reported in 2011 by Jason Arroyo and colleagues determined that miRNAs, besides being encapsulated in exosomes, could also be found associated with protein complexes, specifically Argonaute2, a member of RISC [59]. This discovery raised the possibility that circulating miRNAs could still have a functional role. In support of this, exosomes secreted by lung tumor cells were shown to have a different miRNA composition than the miRNA signature from exosomes derived from normal non-malignant cells [60]. Tumor secreted exosomes, for instance, contained oncogenic miRNAs such as, miR-21, miR-27b, and miR-29a [60]. In addition to repressing target genes in cells that took up the miRNA-loaded exosomes, Croce and colleagues demonstrated that exosomal miR-21 and miR-29a can bind to toll-like receptors (TLRs) and act similar to hormones to trigger inflammatory responses [60]. This specific aspect of miRNA biology, previously unknown to science, suggested the possibility that miRNAs could act as regulators of the tumor microenvironment. In 2014, Sonia Melo and colleagues provided further evidence supporting this hypothesis showing that miRNAs present in breast cancer exosomes can enter normal cells and induce transcriptome alterations that lead to tumor formation [61]. Furthermore, the authors showed that breast cancer secreted exosomes contain the cellular machinery necessary to process pre-miRNAs into mature miRNAs demonstrating for the first time cell independent miRNA biogenesis [61].

## 5. MiRNA Transition into Therapy

### 5.1. Diagnosis, Detection and Prognosis

One of the most challenging aspects of the fight against cancer is to detect cancer accurately and early. For this purpose, multiple research groups around the world are focusing on the identification of potential biomarkers that could be used not only to detect cancer but also to obtain better prognostic values to classify patients both in terms of the mutations and potential response to therapies.

In the case of miRNAs, in 2004 the potential clinical utility of *let-7* as a prognostic tool in human lung cancers was communicated [62]. The Takamizawa group described an inverse correlation between postoperative survival in patients with lung cancer and *let-7* levels [62]. The following year Calin and colleagues identified a 13 miRNA signature that could distinguish patients with CLL and determined a link with disease prognosis and progression [63]. Around the same time, Jun Lu and colleagues determined that it was possible to classify cancers according to their developmental lineage and stage by comparing only miRNA expression profiles [64]. Similarly, in 2008 a pan-cancer miRNA signature was identified using The Cancer Genome Atlas miRNA sequencing data from 12 types of tumors. Interestingly, the authors describe a pan-tumor miRNA signature consisting of a miRNA superfamily that includes seven miRNA families targeting tumor suppressive genes using a GUGC core motif [65]. These findings further highlight the potential of miRNAs as biomarkers for cancer.

Many applications have emerged from the discovery of nucleic acids in circulation [66]. In 2008, reports from independent studies showed that miRNAs could also be found in circulation [55,56,57,58], thus opening a window to develop novel approaches to detect cancer through miRNAs in body fluids. In a proof of principle study, Patrick Mitchell and colleagues, reported that miRNAs are highly stable in serum and provided direct evidence that miRNAs can indeed enter the circulation when originated from tumors of non-hematopoietic origin [55]. Such characteristics make miRNAs particularly appealing for the development of new diagnostic tools along with minimally invasive biopsies. Indeed, miRNAs in both tumor tissues and plasma were shown to harbor predictive, diagnostic and prognostic potential. In this study, the miRNAs signature could predict lung cancer development and aggressiveness even 1–2 years before diagnosis using computed tomography [67]. The diagnostic value of miRNAs has gained additional support from multiple findings, suggesting potential functional roles of circulating miRNAs [60,61].

Besides the miRNA signatures, the mutational status of miRNA binding sites in their protein coding targets can also be regarded as a diagnostic tool. Lena Chin, a graduate student at the time in the Slack laboratory, identified a *KRAS* variant allele that harbors a single nucleotide change (SNP) in a region of the 3′ UTR that contains a *let-7* binding site [68]. Patients carrying this mutant allele of *KRAS* had a higher risk of developing non-small cell lung cancer (NSCLC) [68]. This initial KRAS SNP has since been found and validated as a marker of risk in multiple tumor types, including colorectal [69,70], head and neck squamous cell carcinoma [71,72], endometrial cancer [73], breast and ovarian cancer [74,75] and others. The finding that mutations in miRNA binding sites could affect cancer etiology and disease development represents a new paradigm in the field that until now focused on identifying mutations only in the coding region of genes.

### 5.2. MiRNAs as Therapeutic Agents

MiRNA aberrant expression in cancer is supported by strong evidence, and depending on the status and function of a specific miRNA the therapeutic strategy could be divided into two main classes: inhibition or replacement.

From a historical standpoint, miRNA inhibition was the first approach used to explore the potential of miRNAs in cancer therapy. It started by an observation in Zamore’s group in 2004, in which they showed that it was possible to inhibit *let-7* function in *C. elegans* by introducing a 2′*-O-*methyl oligonucleotide, an RNA analogue, complementary to *let-7* hence acting as an antisense inhibitor [76]. The following year, Jan Krützfeldt’s team successfully tested the potential of miRNA antagonists (termed antagomiRs) in mice and showed that antagomiRs could be used to block endogenous miRNA activity [77]. For instance, delivery of an antagomiR targeting miR-122, a highly abundant miRNA in liver involved in fatty-acid metabolism and hepatitis C virus replication, resulted in de-repression of miR-122 target genes in the liver of mice [77]. In 2008, miR-122 antagomiRs were further validated in non-human primates [78]. In this case, Joacim Elmén and colleagues used an RNA analogue formulated with locked-nucleic-acid (LNA) modified nucleotides [78]. The authors showed that the LNA-antagomiR was able to antagonize miR-122 efficiently and in a dose dependent manner, leading to decreased levels of cholesterol in plasma [78]. Interestingly, the LNA-antagomiR was highly stable in plasma and no signs of toxicity associated with the LNA modification were observed. Functionally, the clinically adaptable miravirsen (SPC3649), an antagomiR targeting miR-122, is able to bind to the stem loop of miR-122 precursors hence blocking both Dicer and Drosha processing [79]. Miravirsen became the first miRNA-targeting drug that entered clinical trials in 2010 and is currently in phase II to treat patients with chronic hepatitis C (ClinicalTrials.gov Identifier: NCT02031133).

Although early research in the antagomiR field was done focusing in liver disease, the experience gained from such studies served as the foundation for the development of oncomiR inhibitors as an emerging therapeutic approach to control cancer. In 2010, Weinberg and colleagues showed for the first time that therapeutically targeting an oncomiR has clinical utility. The Weinberg group showed that an antagomiR targeting the pro-metastatic miR-10b could antagonize metastasis in a murine model of breast cancer [80]. Similarly, in a recent publication, the Slack group introduced a novel strategy to efficiently inhibit miR-155, an oncomiR in a murine model of lymphoma, by using a peptide nucleic acid antagomiR attached to a pH-induced transmembrane structure (pHLIP) [81]. This delivery method takes advantage of the acidic environment of tumors, and overcomes some of the main challenges of antagomiR delivery by not being cleared by the liver and entering cells independently of endocytosis [81]. Various modified antagomiRs are being developed and are currently in preclinical phase showing promising results and are predicted to have a broad impact on the field of oncomiR inhibition.

The second strategy of miRNA therapeutics is to use miRNAs as a therapeutic agent as a replacement strategy. Ectopic replacement of tumor suppressive miRNAs aims to restore the miRNA level in cells and tissues with reduced miRNA expression with the ultimate goal of blocking cellular pathways that drive oncogenesis. One mechanism to deliver miRNAs into cells is through the use of viral vehicles that carry the primary miRNA gene and exploit the cellular machinery to transcribe a pri-miRNA that is further processed into its mature form. Other approaches to deliver miRNAs, that do not require processing to function, use miRNA synthetic mimics, which resemble the mature miRNA duplex (similar to siRNAs) and thus are recognized by RISC [82].

In 2008, in a proof of principle study, Aurora Esquela-Kerscher and colleagues demonstrated for the first time that restoration of *let-7b* was able to inhibit lung cancer *in vivo* in an orthotopic mouse lung cancer model expressing *Kras^G12D^*, an activating mutation for *Kras*. This work provided strong evidence that *let-7b* works as a tumor suppressor in the lung and demonstrated the potential of *let-7b* as a potential miRNA-based therapeutic to treat lung cancer [83]. In 2009, Janaiah Kota and colleagues determined that restoration of miR-26a, a miRNA that is strongly downregulated in liver tumors, via systemic delivery mediated by an adeno-associated virus (AAV) suppresses tumor formation in a Myc-induced mouse model of liver carcinoma [84]. Interestingly, the fact that miR-26a does not target *MYC* for repression suggests that miRNAs could be used as a therapy for cancer even if they do not target the initiating oncogene [84]. In 2010, Jason Wiggins and colleagues tested miR-34a, a key regulator of tumor suppression [39,40,41,42,43], *in vivo* using a synthetic mimic and a lipid vehicle and showed that ectopic miR-34a blocks tumor growth in a xenograft model of lung cancer [85]. Remarkably, this study provided proof-of-principle for the successful delivery of miRNA mimics systemically obviating the use of viral vehicles. This was further supported by follow up studies conducted by the Slack group. In 2011, miR-34a was delivered using a neutral lipid emulsion in a murine model of non-small cell lung cancer (NSCLC) driven by *Kras^G12D^*, which lead to a decrease in tumor burden [86]. Similarly, in 2012, Andrea Kasinski, working in the Slack laboratory, demonstrated that lentiviral delivered miR-34a prevents both initiation and cancer progression in an aggressive mouse model of NSCLC that recapitulate the most common mutations in human lung cancers, loss of p53 and activation of KRAS [87].

It is thus no surprise, based on the pre-clinical data using miR-34a, that in April of 2013, miR-34a was the first miRNA that reached phase I clinical trials (ClinicalTrials.gov Identifier: NCT01829971) in patients with liver cancer sponsored by Mirna Therapeutics (Austin, TX, USA). Mirna Therapeutics’ MRX34, is a miR-34a mimic encapsulated in a liposome vesicle developed by Marina Biotech (Bothell, WA, USA) [88]. In April of 2014 the phase I safety [89] data was announced, showing promising results. The trial is currently administering the fifth level dose at 70 mg/m^2^ and the maximum tolerated dose has not yet been reached [89]. It is expected that phase I will be completed by the end of 2015. There are other companies such as Regulus Therapeutics (San Diego, CA, USA), Sanofi (Paris, France), miRagen Therapeutics (Boulder, CO, USA) that are also conducting preclinical studies and have identified promising miRNAs candidates that are currently being developed. 

Recent studies showed that it was possible to further enhance miR-34a’s anti tumorigenic activity using a combinatorial approach. Such combinatorial therapies include combinations of different miRNAs [90], miRNA-siRNA combinations [91] and miRNAs-targeted drug combinations [92]. Collectively these studies have opened a window to the new exciting field of combinatorial therapeutics with miRNAs that will likely further facilitate the transition of miRNAs into the clinic. 

## 6. Closing Remarks

Many pioneer studies over the past twenty-two years have established the importance of miRNAs in normal cellular homeostasis and repeatedly have demonstrated the potential of miRNAs as cancer therapeutics. One point that emerges from the brief history of miRNAs is that the detailed study of their biology will lead to new findings of importance to understand cancer and potential strategies to control it. Indeed, notable progress has been made in the last 13 years since the discovery of miRNA’s role in cancer. However, miRNA-based therapeutics and diagnoses are still in their infancy and there are important challenges that the scientific community still needs to address.

Currently one of the biggest challenges for miRNA advancement into the clinic is delivery. Efficient delivery of miRNAs faces various barriers, such as delivery-associated toxicity, poor transfection, systemic clearance, poorly understood biodistribution, degradation in circulation, immune response, and endosomal sequestration [93]. We fully acknowledge that remarkable strategies for miRNA delivery are being developed to overcome these obstacles and facilitate miRNA transport. We anticipate that rational design of safe and efficient new generation miRNA carriers will be instrumental for the clinical translation of miRNAs.

Another challenge that requires further study is the safety profile of miRNAs. It is expected that the results from the first miRNA cancer treatment in early clinical trials will provide the ultimate answer to that concern; and yet, preliminary evidence has not shown evidence of negative side effects or problems with dose tolerance [89]. The history of miRNAs is particularly inspiring in showing the importance of basic research, which in this case, has proven to be relevant in the development of new treatments for cancer.
